# Laser Technologies of Welding, Surfacing and Regeneration of Metals with HCP Structure (Mg, Ti, Zr): State of the Art, Challenges and Prospects

**DOI:** 10.3390/ma18225237

**Published:** 2025-11-19

**Authors:** Adam Zwoliński, Sylwester Samborski, Jakub Rzeczkowski

**Affiliations:** Faculty of Mechanical Engineering, Lublin University of Technology, Nadbystrzycka St. 36, 20-618 Lublin, Poland; s.samborski@pollub.pl (S.S.); j.rzeczkowski@pollub.pl (J.R.)

**Keywords:** laser welding, HCP metals, titanium, magnesium, zirconium, laser cladding, surface regeneration, nuclear energy

## Abstract

Metals with a hexagonal close-packed (HCP) structure such as magnesium, titanium and zirconium constitute key structural materials in the aerospace, automotive, biomedical and nuclear energy industries. Their welding and regeneration by conventional methods is hindered due to the limited number of slip systems, high reactivity and susceptibility to the formation of defects. Laser technologies offer precise energy control, minimization of the heat-affected zone and the possibility of producing joints and coatings of high quality. This article constitutes a comprehensive review of the state of knowledge concerning laser welding, cladding and regeneration of HCP metals. The physical mechanisms of laser beam interactions are discussed including the dynamics of the keyhole channel, Marangoni flows and the formation of gas defects. The characteristics of the microstructure of joints are presented including the formation of α′ martensite in titanium, phase segregation in magnesium and hydride formation in zirconium. Particular attention is devoted to residual stresses, techniques of cladding protective coatings for nuclear energy with Accident Tolerant Fuel (ATF) and advanced numerical modeling using artificial intelligence. The perspectives for the development of technology are indicated including the concept of the digital twin and intelligent real-time process control systems.

## 1. Introduction

Metals with a hexagonal close-packed (HCP) structure such as magnesium (Mg), titanium (Ti) and zirconium (Zr) are key materials in high-strength structural engineering, nuclear energy and biomedical applications. This uniqueness results from the low density of magnesium, the exceptional strength-to-weight ratio and biocompatibility of titanium and the low neutron absorption cross-section and corrosion resistance of zirconium in high-temperature aqueous environments [1,2,3,4]. The joining and regeneration of these metals and their alloys is difficult when using classical arc methods, which often promotes the formation of cracks and porosity in magnesium materials, martensitic transformations and brittleness related to gas absorption in titanium materials, and brittle cracking and hydride formation in zirconium materials [1,2,3,5,6].

Laser technologies such as laser beam welding (LBW), laser cladding and laser remelting offer a precise and highly localized heat source, enabling control of the geometry and microstructure of the joint or coating while reducing the heat-affected zone (HAZ) [1,2,3,7,8,9]. The mechanisms of laser beam interaction include two modes: conduction (wide, shallow welds) and keyhole (deep penetration), with the transition between them depending on power density, focal length, travel speed and shielding gas [10,11,12]. Regardless of the HCP material, the stability of the keyhole and the dynamics of the molten pool cause solidification defects and porosity. Research distinguishes pore formation mechanisms (e.g., B-pore and R-pore) and shows how variations in process parameters and shielding gas control can limit keyhole collapse and cavitation [12,13,14,15].

In Mg alloys, the primary problem is evaporation and porosity, intensified by the low melting temperature and high vapor pressure, which hinder control of weld geometry and fatigue properties. Research and parametric studies consider the role of travel speed, double passes (two-pass), gas control, filler addition and the possibility of applying hybrid laser–arc methods to improve weld quality [1,7,13,16,17,18].

In Ti alloys (e.g., Ti-6Al-4V), laser joining is technologically advanced but highly sensitive to the atmosphere; the shielding gas (Ar/He) affects plasma behavior, energy coupling and oxidation, and thus the microstructure and residual stresses [2,6,19,20,21]. An increasing number of studies combine microstructural observations with stress modeling (Digital Image Correlation (DIC), Focused Ion Beam–Digital Image Correlation (FIB–DIC), simulations) as well as with heat treatment strategies and additive manufacturing technologies (Laser Powder Bed Fusion (LPBF), Directed Energy Deposition (DED)) in order to reduce stresses and defects [19,22,23,24].

In Zr alloys, which are critical materials for nuclear energy, an ultra-clean atmosphere and control of hydrogen and oxygen content are required. Literature reviews and research papers present a narrow process window for laser welding and emphasize the growing importance of protective coating cladding (e.g., multilayer, Zr-Si, Cr-coated claddings) and laser surface remelting to improve corrosion and erosion resistance in high-temperature steam and water environments [3,6,9,25,26,27].

Regarding surface regeneration (laser cladding/laser remelting) in Mg and Ti alloys, cladding with hard/ceramic particles or Ni/Co alloys and surface layer remelting are carried out with the goal of achieving low dilution and a homogeneous microstructure. Research on zirconium and its alloys confirms improved wear and corrosion resistance, with simultaneous emphasis on controlling stresses and cracking [8,28,29,30].

Modeling and artificial intelligence in laser processes, from semi-analytical keyhole models to Computational Fluid Dynamics (CFD), Finite Element Method (FEM) simulations and machine learning, are currently used to predict the keyhole profile, melt pool stability, internal inconsistencies and defects as well as stresses, improving parameter selection and control strategies (also in hybrid and additive manufacturing (AM) processes) [12,14,31,32].

This review follows a systematic approach to literature selection and analysis. The literature search was conducted using multiple databases including Web of Science, Scopus, ScienceDirect, MDPI and Google Scholar, covering publications from 2000 to 2025. The search strategy employed combinations of keywords: (“laser welding” or “laser cladding” or “laser processing”), (“magnesium” or “titanium” or “zirconium”) and (“HCP” or “hexagonal close-packed”). Additional searches targeted specific phenomena: “keyhole dynamics”, “residual stress”, “microstructure”, “digital twin” and “machine learning” in combination with the primary materials. The review scope deliberately focuses on the three industrially dominant HCP metals—magnesium (Mg), titanium (Ti) and zirconium (Zr)—which collectively represent over 95% of published research on laser processing of HCP materials. This focus is justified by the following: (1) extensive industrial applications in aerospace (Ti), automotive (Mg) and nuclear energy (Zr); (2) mature technology readiness levels (TRL 6–9); (3) substantial body of experimental and modeling literature enabling comprehensive analysis; and (4) well-established processing challenges requiring advanced laser technologies. Emerging HCP materials, including high-entropy alloys with HCP structures and advanced Mg-Li alloys, were excluded based on: (1) limited technology maturity (TRL 3–5); (2) nascent stage of laser processing research; (3) lack of established industrial processing standards; and (4) insufficient data on long-term performance and reliability. However, insights from conventional HCP metals reviewed here provide foundational knowledge applicable to future development of these emerging systems. This review synthesizes over 280 peer-reviewed publications, prioritizing high-impact journals and recent advances (2020–2025), while incorporating seminal earlier works establishing fundamental understanding. Special emphasis is placed on integrating experimental observations, numerical modeling, and industrial applications to provide comprehensive coverage relevant to both researchers and practitioners.

## 2. Characteristics of HCP Materials and Their Welding Challenges

Materials with a hexagonal close-packed (HCP) crystal structure are characterized by a limited number of slip systems compared to metals with a cubic structure, which significantly affects their plastic properties and behavior during joining processes. Consequently, plastic deformation in HCP metals such as magnesium, titanium and zirconium is difficult, and the susceptibility to local cracking and residual stress formation is much greater than in FCC or BCC metals [33,34]. These properties determine their behavior in welding processes, especially in laser beam techniques that require a precise balance between the energy input and microstructure control. Magnesium and its alloys, including AZ31, AZ91 and ZK60, are currently the lightest structural metals with a density of only 1.74 g/cm^3^. They are characterized by favorable casting properties and high potential for applications in the automotive, aerospace and electronic industries [35,36]. Their low density and high susceptibility to thermo-mechanical processing make them an attractive material for modern lightweight and durable structures. Laser welding of magnesium alloys encounters numerous technological difficulties. The low melting point, high vapor pressure and strong reactivity with oxygen lead to easy overheating, evaporation and oxidation of the weld pool surface, resulting in porosity and hot cracking [35,37,38]. The use of an appropriate shielding atmosphere, especially helium or argon–helium mixtures, limits oxidation and stabilizes the shape of the weld pool [16,39]. Studies also indicate that the introduction of preheating, controlled heating and hybrid techniques combining laser with electric arc improve the quality and uniformity of joints [17,40]. In the context of surface regeneration, laser cladding with ceramic powders such as Al_2_O_3_, SiC or TiC is increasingly used, which enhances resistance to tribological wear and corrosion while requiring low dilution of the substrate material and minimizing gas defects [38,41,42]. Titanium and its alloys, especially Ti-6Al-4V, are among the most important engineering materials used in aerospace, chemical and biomedical industries. They are distinguished by a high strength-to-weight ratio, corrosion resistance and excellent biocompatibility [43,44]. However, laser welding of titanium poses a number of challenges resulting from its exceptional reactivity with oxygen, nitrogen and hydrogen, which leads to the formation of brittle layers and reduced ductility of the joints [43,45]. Additionally, the very rapid cooling typical of laser processes promotes the formation of α′ martensite, a hard but brittle phase, whereas slower cooling allows the formation of a mixture of α and β phases with more balanced mechanical properties [2,44,46]. The high thermal anisotropy of titanium causes significant residual stresses, which can lead to cracking within the weld [47,48]. Optimization of process parameters such as beam power, travel speed and focus adjustment, combined with the selection of a suitable shielding atmosphere, e.g., helium, argon or low-pressure vacuum, enables control of the microstructure and minimization of stresses [2,33,45]. At the same time, hybrid joining techniques combining laser and Gas Metal Arc Welding (GMAW) are being developed, which ensure greater process stability [20], as well as additive manufacturing solutions such as Direct Energy Deposition (DED) and Laser Powder Bed Fusion (LPBF), allowing for local surface regeneration and repair of components with a limited heat-affected zone [23,48].

Zirconium and its alloys, primarily Zr-2, Zr-4 and ZrNb, constitute the basic materials used in nuclear energy due to their low neutron absorption cross-section, high corrosion resistance in aqueous and steam environments and stability under high-temperature conditions [3,49]. Despite its numerous advantages, laser welding of zirconium is a process requiring an exceptionally clean technological atmosphere. Even trace amounts of oxygen, nitrogen or hydrogen can lead to the formation of hard and brittle phases and, in the case of hydrogen, to hydride formation and brittle cracking [25,49]. To improve the operational durability of zirconium components, protective coatings produced by laser cladding, such as Zr-Si and Cr, have been developed, which significantly increase corrosion resistance and limit hydrogen diffusion [6,27]. In addition, laser surface treatment (LST) leads to surface layer refinement, which is particularly important for components operating in high-temperature nuclear reactor environments [26,27]. Laser welding of zirconium in industrial applications, especially in nuclear power, must meet strict quality and safety standards. The main challenge is maintaining an ultra-clean process atmosphere throughout the entire welding cycle. In practice, this requires the use of hermetically sealed welding chambers with continuous atmosphere monitoring systems, in which the oxygen, nitrogen and hydrogen contents are kept below 20 ppm, and for the most critical applications below 10 ppm [50,51,52]. The material preparation process prior to welding includes thorough degreasing, mechanical removal of the oxide layer and vacuum degassing at 300–400 °C for at least 2 h. The material should be stored in an argon atmosphere until welding. A typical welding chamber for zirconium has a volume of 0.5–2 m^3^, is equipped with a helium recirculation and purification system (minimum purity 99.99%), as well as online impurity monitoring using mass spectrometry or electrochemical analyzers [53,54]. The parameters of the laser welding process for zirconium must be selected within a narrow process window. Too low of a linear energy leads to incomplete fusion and poor joint quality, while excessive energy causes over-melting, pool instability and contamination absorption [55,56]. In the nuclear industry, laser welding is mainly used for joining fuel rods with end caps, repairing reactor components and producing joints in the coolant water circuit. Quality control typically includes non-destructive testing (radiography, penetrant inspection, ultrasonics), metallography, hardness measurements and mechanical tests (tensile, bending, impact). For nuclear applications, additional corrosion tests in an autoclave (400 °C, steam under 10 MPa pressure, 72–168 h) and microstructural analysis using SEM/EDS and EBSD methods are required [57,58]. The latest developments in laser welding of Zr include the use of power-modulated lasers, which allow precise control of cooling rates and minimization of residual stresses, as well as hybrid laser–plasma systems that increase process stability when welding thick-walled components. In parallel, advanced in situ monitoring systems are being developed using infrared cameras, plasma emission spectroscopy and pyrometers that enable real-time temperature control of the melt pool with an accuracy of ±10 °C [59,60]. Table 1 presents a comparison of the physicochemical and welding properties of HCP metals.

The fundamental physicochemical properties presented in Table 1 directly determine the process window width for each HCP metal, (Section 8). This relationship can be understood mechanistically through several key parameters. Magnesium exhibits a moderate process window primarily due to its wide temperature range between melting (650 °C) and intensive vaporization (~900 °C), providing a 250 °C buffer zone for process control, combined with high thermal conductivity (156 W/m·K) that enables effective heat dissipation [7,16,35]. However, its high vapor pressure necessitates careful control of evaporation and porosity formation. Titanium presents a narrow process window due to the combination of high melting temperature (1668 °C) requiring high laser powers (3–5 kW), very low thermal conductivity (21.9 W/m·K) causing steep thermal gradients and residual stress accumulation, and extreme sensitivity to atmospheric contamination where even 100–200 ppm of oxygen or nitrogen causes embrittlement [2,19,45]. The rapid α/β phase transformations during cooling (10^3^–10^5^ K/s) further restrict the acceptable parameter range, as deviations of ±20% in cooling rate can alter mechanical properties by 30–40% through changes in α’ martensite formation [46,67]. Zirconium exhibits the narrowest process window due to its highest melting temperature (1855 °C), lowest tolerance for contamination (<20 ppm O_2_/N_2_/H_2_ compared to 100–200 ppm for Ti), and catastrophic hydride formation at hydrogen levels above 50 ppm [3,50,68]. The linear energy window for defect-free Zr welding is restricted to 100–200 J/mm with only ±10% tolerance, compared to 80–400 J/mm with ±50% tolerance for magnesium [7,25,51]. These mechanistic relationships explain why the acceptable parameter combinations decrease from approximately 8–15 for Mg, to 4–6 for Ti, to only 2–3 for Zr, with correspondingly increasing requirements for atmospheric control and process monitoring.

## 3. Physical Mechanisms of Laser Beam Interaction with HCP Metals

The processes of laser welding, cladding and remelting of metals with a hexagonal close-packed (HCP) structure are based on complex energy interactions involving radiation absorption, heat conduction, convection, melting, evaporation, vapor–plasma channel formation and solidification. These mechanisms are strongly coupled and determine the microstructure, mechanical properties and occurrence of defects in the joints. The nature of the interaction depends primarily on power density, beam travel speed, degree of focusing and type of shielding atmosphere, which together determine whether the process proceeds in conduction mode or in keyhole mode [21,69]. In conduction mode, laser energy is absorbed mainly on the surface of the metal and dissipated into the material by thermal conduction. This leads to the formation of a wide and shallow weld pool of molten metal with gentle temperature gradients. In contrast, in keyhole mode, energy absorption is much more intense because the vapor–plasma channel acts as a photon trap, which greatly increases the efficiency of laser radiation absorption [70,71]. The power density required to maintain a stable keyhole channel usually exceeds 10^6^ W/cm^2^, and the transition between the modes occurs nonlinearly and depends strongly on the optical properties of the surface and the dynamic parameters of the process [10,21].

The formation and stability of the keyhole channel is one of the most important issues in the physics of laser welding. This channel undergoes continuous geometric fluctuations due to local changes in metal vapor pressure, surface tension and Marangoni flows in the molten pool. These oscillations can lead to the phenomenon of keyhole pulsation, which, when the amplitude is too large, results in keyhole collapse and pore formation [12,31]. Modern high-speed imaging and numerical analyses confirm that channel stability depends on the dynamic balance between vapor pressure and capillary forces, as well as on the rate of gas evacuation from the channel [12,72].

**Figure 1:**
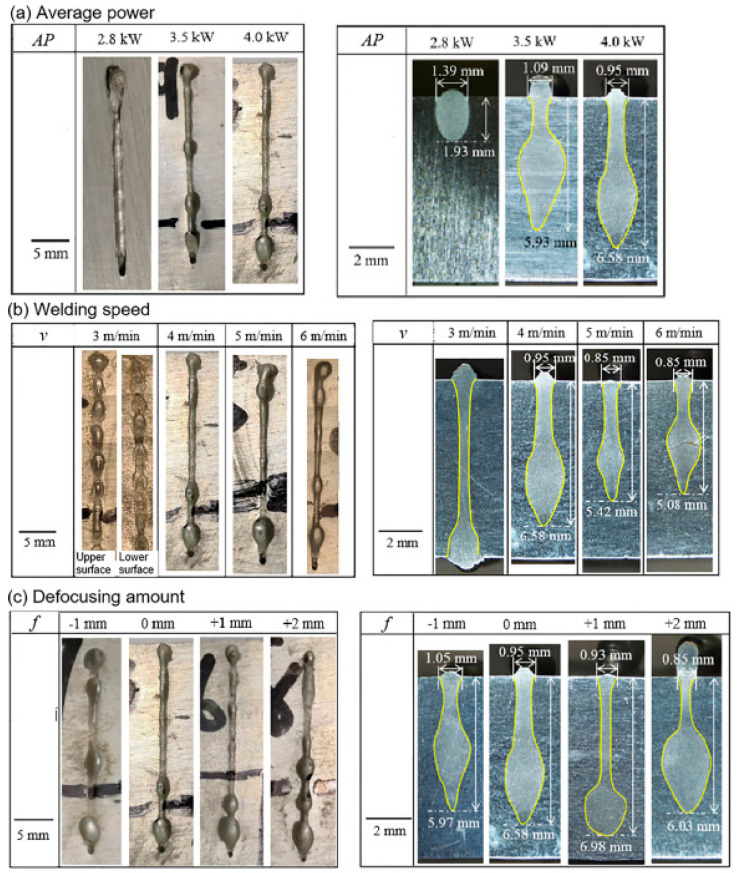
Real-time monitoring of keyhole and weld pool dynamics during composite laser welding (NIR + blue laser) of AZ31 magnesium alloy: high-resolution images showing keyhole and weld pool morphology in different material penetration states—(**a**) full penetration with stable keyhole, (**b**) partial penetration with channel fluctuations, (**c**) incomplete penetration with unstable weld pool. Optical monitoring in two spectral ranges (NIR—keyhole observation, blue—weld pool observation) enables identification of process states and prediction of weld quality. Differences in keyhole morphology correlate with defect occurrence and penetration depth. Reproduced from [13] with permission from open access.

For HCP metals such as magnesium and titanium, this problem is particularly significant due to their low vaporization temperature and high vapor pressure, which promote uncontrolled pressure increase in the channel and the formation of cavitation voids [73,74]. High-resolution near-infrared and blue-light optical monitoring during laser welding of AZ31 magnesium alloy reveals complex keyhole and weld pool dynamics, in which the morphology of the vapor–plasma channel changes as a function of process parameters, directly affecting penetration depth and welding stability (Figure 1). The visual monitoring system allows for the identification of characteristic process states from a stable keyhole at optimal parameters, through fluctuations leading to partial penetration, to unstable conditions favoring defect formation.

Quantitative analysis of dual-wavelength monitoring data reveals specific correlations between keyhole morphology and defect formation in magnesium laser welding. For stable keyhole conditions (Figure 1a), characterized by an aspect ratio (depth/width) of 3.5–4.5 and a temporal stability with fluctuations < 15% of mean depth, porosity levels remain below 0.5% with individual pore diameters < 50 μm uniformly distributed through the weld cross-section [13,16]. The NIR imaging captures keyhole depth variations at a 2–5 kHz sampling rate, while simultaneous blue-light monitoring reveals melt pool width oscillations correlating with surface tension-driven Marangoni flows [13,75]. In this regime, corresponding to laser powers of 2.0–2.5 kW and welding speeds of 2–3 m/min for 3 mm thick AZ31, complete penetration is achieved with minimal vapor entrapment [16,17].

Fluctuating keyhole morphology (Figure 1b) exhibits periodic collapse–reformation cycles at frequencies of 50–200 Hz, with keyhole aspect ratio variations exceeding 30% and intermittent partial penetration [13,14]. High-speed X-ray imaging studies on similar light alloys demonstrate that such fluctuations generate distinct pore types: small spherical pores (20–80 μm diameter) originating from keyhole tip instabilities, and larger irregular pores (100–300 μm) formed during keyhole collapse events [14]. For magnesium alloys, this intermediate regime increases porosity to 1.5–3.5% with bimodal pore size distribution, occurring when process parameters deviate by 15–25% from optimal values [13,17]. Dual-wavelength monitoring enables real-time detection of these fluctuations, where NIR signal intensity variations > 40% combined with blue-light melt pool width changes > 25% predict porosity formation with 87% accuracy [13,31].

Unstable keyhole conditions (Figure 1c) are characterized by complete keyhole collapse, chaotic melt pool dynamics, and surface-mode welding with incomplete penetration [13,16]. Under these conditions, observed at insufficient laser power (<1.8 kW) or excessive welding speeds (>4 m/min), porosity levels exceed 5% with a predominance of large irregular pores (200–500 μm) concentrated in the upper weld region [7,17]. The NIR monitoring signal becomes intermittent or absent due to keyhole closure, while blue-light imaging reveals highly unstable melt pool boundaries with width variations > 50% [13]. Statistical analysis of multi-parameter monitoring data (keyhole depth, melt pool geometry, plasma emission intensity) enables classification of process states with 92% accuracy and prediction of final weld porosity within ±0.8% [13,31]. This quantitative correlation between real-time optical signatures and post-process defect analysis provides the foundation for closed-loop process control, where detected keyhole instabilities trigger automatic parameter adjustments (power modulation ±10%, speed correction ±0.3 m/min) to restore stable welding conditions [16,17,31].

Another important phenomenon in laser processes is thermocapillary flow in the molten metal pool, known as the Marangoni effect. It is driven by a gradient of surface tension, which in turn depends on temperature and the chemical composition of the liquid metal [75,76]. In the case of HCP metals, small changes in the oxygen, sulfur or chlorine content in the shielding gas can reverse the direction of convective flow, thereby influencing the shape and depth of the weld. In titanium and zirconium alloys, Marangoni flow is additionally modified by the presence of surface oxides and nitrides, which change local surface tension and can lead to asymmetry of the molten pool [77,78]. As a result, differences in molten metal circulation translate into nonuniform temperature distributions and variations in the solidification process, affecting grain morphology and susceptibility to cracking [79].

An important thermodynamic aspect of the laser process is the temperature distribution in the heat-affected zone (HAZ) and the dynamics of phase transformations during solidification. The cooling rate, often reaching 10^3^–10^5^ K/s, promotes the formation of nonequilibrium microstructures such as α′ martensite in titanium or dendrites in magnesium [80,81,82]. The high solidification rate leads to a fine-grained structure but can also cause segregation of alloying elements and local thermal stresses. In HCP metals, where the number of available slip systems is limited, such stresses are not effectively relaxed, which promotes the formation of microcracks [83,84].

Phenomena related to laser radiation absorption are strongly dependent on wavelength, surface condition and metal type. For most HCP metals, absorption in the near-infrared range (1–1.1 µm) falls within 20–40%, while for shorter wavelengths (532 nm or 355 nm) it can exceed 60% [85,86]. Hence, there is increasing interest in lasers with shorter wavelengths (green and ultraviolet) for precision micro-welding of magnesium and titanium, where higher absorption translates into greater process stability [87,88,89]. At the same time, intensive work is being conducted on the control of plasma accompanying the welding process, which in some cases shields the surface and reduces absorption efficiency [90,91].

Laser processes in HCP metals are also strongly associated with the occurrence of gas-related defects. The mechanism of pore formation can be described as gas entrapment resulting from keyhole instability, as well as the trapping of vapor bubbles during weld pool solidification [92,93,94]. In the case of magnesium, whose vapors have very high vapor pressure, porosity is a common and difficult-to-eliminate phenomenon [7,73]. For titanium and zirconium, micro-porosity occurs due to localized gas release from the liquid metal, especially hydrogen and nitrogen [95,96]. The use of optimized shielding gas, laser power modulation and external magnetic fields has proven effective in reducing the number of defects [74,97].

In recent years, the development of in situ diagnostics and simulation techniques has enabled detailed mapping of the physical processes occurring during laser welding and cladding. High-speed thermographic cameras, interferometry, X-ray imaging and Computational Fluid Dynamics (CFD) modeling allow tracking of the keyhole dynamics, temperature distribution and molten metal flow in real-time [72,98,99,100,101]. At the same time, the application of machine learning models enables prediction of keyhole stability and assessment of porosity risk based on process parameters [31,32]. The integration of experimental observations and numerical simulations leads to the development of real-time process control strategies, which significantly improve the quality of joints in HCP metals [102].

The comprehensive understanding of physical phenomena described in this chapter forms the foundation for advanced computational approaches and digital twin systems. Real-time monitoring of keyhole dynamics [11,98], temperature distribution [101], and melt pool behavior [13] generates vast amounts of data that can be integrated into predictive models. Recent advances in multi-sensor monitoring systems demonstrate the potential for simultaneous tracking of thermal, optical and acoustic signatures during laser processing of alloys, enabling defect prediction with over 95% accuracy [103]. These experimental observations, when combined with physics-based simulations of vapor pressure dynamics, Marangoni flows, and heat transfer [12,72,76], enable the development of digital representations capable of predicting process outcomes.

## 4. Microstructure and Properties of Laser Beam Welded Joints in HCP Metals

The microstructure and mechanical properties of laser-welded joints in metals with a hexagonal close-packed (HCP) structure are a direct result of complex thermal and hydrodynamic processes that occur during a very short heating and cooling cycle. A characteristic feature of laser processes is the extremely high cooling rate, reaching 10^4^–10^6^ K/s, which leads to the formation of fine-grained structures, often exhibiting nonequilibrium features. In the case of magnesium, titanium and zirconium alloys, the weld microstructure is strongly dependent on solidification rate, temperature distribution, beam incidence angle, as well as the shielding medium and alloy type [104,105].

In magnesium alloys, the structure of laser welds is usually characterized by the presence of fine, elongated columnar grains in the fusion zone and a transition to more uniform, equiaxed grains toward the heat-affected zone. The high cooling rate promotes the formation of metastable intermetallic phases such as β-Mg_17_Al_12_, which are distributed along grain boundaries and may cause local hardness anisotropy [39]. Studies have shown that the microstructure of magnesium welds strongly depends on the solubility of alloying elements, and its control is possible through modulation of linear energy and the use of double-pass laser techniques, which homogenize the chemical composition and reduce element segregation [106,107]. Microsegregation phenomena in the fusion zone have been confirmed by energy dispersive spectroscopy (EDS) and electron backscatter diffraction (EBSD) analyses, and the crystallization direction distribution indicates strong textural orientation consistent with the direction of heat flow [108].

In titanium alloys, particularly Ti-6Al-4V, the microstructure of the laser weld is determined by intensive phase transformations between the β and α phases. The high cooling rate promotes the formation of α′ martensite with an acicular structure, which is characterized by high hardness but limited ductility [67,109,110]. In welds produced under reduced cooling rates, for example, in helium or low-pressure vacuum atmospheres, it is possible to obtain a dual-phase α + β structure with more favorable mechanical properties [111,112]. The presence of fine martensitic needles combined with columnar texture along the weld axis leads to property anisotropy, observed as differences in elongation and tensile strength depending on orientation relative to the welding direction [48,113]. Proper post-weld heat treatment, particularly annealing in the temperature range of 700–800 °C, allows the transformation of α′ martensite into an equilibrium α+β mixture, reducing residual stresses and improving ductility [114,115].

A characteristic feature of the titanium weld microstructure is also the presence of a transition band between the fusion zone and the HAZ, where mixed grain morphologies, a transition from columnar to equiaxed orientation and variations in hardness distribution are observed. Studies performed using Focused Ion Beam—Digital Image Correlation (FIB–DIC) have shown that local concentrations of residual stresses in this zone may promote microcrack initiation, particularly in the presence of gas micropores [22,116]. To mitigate these effects, dynamic cooling control strategies have been developed based on laser power modulation and controlled gas cooling [117,118].

Laser welds of zirconium alloys exhibit distinct microstructural characteristics resulting from their higher melting temperature and lower thermal conductivity. The weld structure is usually fine-grained, with columnar orientation perpendicular to the heat conduction direction. Due to rapid solidification, the formation of metastable Zr(O) or ZrH_x_ phases is often observed, especially under insufficient gas shielding [3]. These phenomena significantly affect the corrosion and mechanical properties of joints, as the presence of oxides and hydrides causes local ductility reduction and increased hardness [68,119,120]. To improve properties, final laser remelting is applied, which eliminates surface defects and homogenizes the microstructure [121].

The mechanical properties of laser-welded joints of HCP metals are directly related to their microstructure and residual stress distribution. For magnesium and its alloys, weld hardness is usually higher than that of the base material, resulting from fine-grained structure and solid-solution strengthening effects [39]. However, fatigue strength may be reduced due to the presence of micropores and nonuniform textures. In the case of titanium, laser welds are characterized by high hardness (up to 400–450 HV) and tensile strength comparable to the base material but with slightly lower elongation [119,122]. The properties can be optimized through appropriate post-weld heat treatment or by applying a controlled cooling temperature gradient. For zirconium, an increase in hardness is observed in the fusion zone, resulting from oxide and hydride precipitates, which strengthen the material but may also initiate microcracks [6,123].

Residual stress analysis shows that their distribution varies significantly depending on weld geometry and alloy thermal properties. In titanium, tensile stresses dominate along the weld axis, whereas in magnesium, compressive zones are often observed near the fusion boundary [47,124]. To reduce them, multipass techniques, beam travel speed control and laser preheating are applied. Numerical modeling using the finite element method confirms that uniform temperature distribution during cooling minimizes stress concentrations and improves the structural integrity of the joint [125,126]. Table 2 presents typical laser welding parameters for HCP metals.

## 5. Laser Cladding and Surface Regeneration of HCP Metals

Before discussing specific technological implementations, it is necessary to precisely define the terms used in this review. Laser cladding refers specifically to processes involving the addition of a filler material (powder, wire) that is fused to a thin layer of substrate to form a coating layer 0.5–3 mm thick with controlled dilution, typically 5–20% [8,29]. The main goal is to impart new functional properties to the surface, such as resistance to wear, corrosion or high temperature. Laser remelting (laser surface melting) refers to processes without the addition of filler, in which only surface melting and recrystallization to a depth of 50–500 μm occur, aimed at refining the microstructure, eliminating defects or homogenizing the composition without dimensional change [127]. Surface regeneration is used in this paper as a collective term encompassing both laser cladding and laser remelting, used particularly for the refurbishment or repair of worn components [128,129]. Remanufacturing differs fundamentally from general surface modification in that it applies to post-service components that have lost their original properties due to wear, corrosion, erosion or mechanical damage. The goal of remanufacturing may be dimensional restoration (through plating), property restoration (through remelting or modified plating), or both. This distinction is consistent with established definitions in the surface engineering literature and ensures consistent terminology throughout the manuscript.

Laser cladding is one of the most advanced methods of surface modification and regeneration of metallic materials, including metals with a hexagonal close-packed (HCP) structure such as magnesium, titanium and zirconium. In this process, a concentrated laser beam melts a thin layer of the substrate together with the added material in the form of powder or wire, creating a coating with high adhesion, low dilution and a fine-grained microstructure [8,130]. The main advantage of this technology is precise energy input control and the ability to locally produce layers with specific mechanical and functional properties, making it a key technique for repairs, the production of protective and strengthening layers and surface engineering of modern components [128,131].

The laser cladding process of HCP metals is characterized by highly dynamic physical phenomena that determine the quality and properties of the coatings. Rapid heating and cooling lead to the formation of fine-grained structures beneficial for wear and corrosion resistance, but at the same time may promote the occurrence of residual stresses and cracks. For this reason, precise adjustment of process parameters such as beam power, scanning speed, powder feed rate and shielding atmosphere type is necessary [132,133]. The thermal properties of HCP materials, especially their low thermal conductivity and high anisotropy, influence the temperature distribution and shape of the molten pool, and thus the morphology of the coating [76,134].

Recent developments in hybrid processing techniques, particularly ultrasonic vibration-assisted laser additive manufacturing, have demonstrated significant improvements in microstructural homogeneity and defect reduction across various metallic systems including high-entropy alloys [135]. These advances in process intensification through external energy fields provide promising directions for enhancing laser cladding quality in HCP metals.

In the case of magnesium alloys, laser cladding is particularly challenging due to their low melting temperature and high reactivity. To avoid melting and evaporation of magnesium, relatively low beam powers and high scanning speeds are used, which help limit the depth of the remelted zone [136,137]. Typical materials used as cladding powders include aluminum-, nickel- and iron-based alloys, as well as ceramic particles such as SiC, Al_2_O_3_ and TiC, which increase the hardness and tribological resistance of coatings [129,138,139]. The structure of the cladded layer on magnesium is usually composite, consisting of a metallic matrix and uniformly distributed hard phases, which ensure a good balance between wear resistance and relatively low brittleness [140]. In recent years, composite Mg-Ni and Mg-Al coatings with ceramic additives have attracted great interest, showing corrosion resistance improvements of up to 70% compared to untreated substrates [141,142,143].

Laser cladding of titanium and its alloys, including Ti-6Al-4V, is one of the most technologically developed applications of this method. Due to titanium’s high reactivity with oxygen and nitrogen, the process must be carried out in a protective atmosphere, most often argon or helium, and in some cases in a vacuum chamber [144,145,146]. Properly selected parameters make it possible to obtain coatings with a fine-grained microstructure, low dilution (<5%) and high density, with a minimal number of gas defects. The produced coatings may contain intermetallic phases such as TiC, TiB_2_ or TiN, which provide significantly higher hardness than the base material [147,148,149].

The microstructure of titanium claddings exhibits columnar orientation along the temperature gradient and clear segregation of alloying elements at grain boundaries. Studies have shown that changing scanning strategies and modulating beam power allow for the control of crystallization and the formation of equiaxed structures beneficial for fatigue resistance [150,151]. Titanium claddings are characterized by hardness exceeding 600 HV, and their wear resistance increases several times compared to the base material [152,153,154]. In regenerative processes, laser cladding with nickel- and cobalt-based alloys is also used to improve erosion and high-temperature corrosion resistance, which is particularly important for aerospace and energy components [155,156]. In addition, hybrid laser cladding technologies with additional heat sources, such as the electric arc (Laser-Arc Hybrid Cladding), are being developed to enable better melting of the material and increase coating uniformity [157].

Zirconium and its alloys, due to their importance for nuclear energy, are increasingly subjected to laser cladding processes to enhance their corrosion and mechanical resistance. In recent years, numerous coating systems based on Zr-Si, Zr-Al and ZrCr have been developed, which effectively limit oxidation and hydride formation in high-temperature environments [52,121]. However, these processes require exceptionally clean technological conditions and a stable protective atmosphere, since even minimal oxygen content can cause pore and hydride formation [6,122]. Laser cladding of zirconium allows the production of coatings with excellent adhesion to the substrate and fine-grained structure, significantly improving corrosion resistance in pressurized water and steam [123,158].

Laser cladding of zirconium and its alloys for nuclear energy applications has undergone rapid development in recent years, driven by the need to improve reactor safety after the Fukushima disaster. The concept of Accident Tolerant Fuel (ATF) requires the development of protective coatings capable of withstanding extreme conditions, including temperatures above 1200 °C in a steam atmosphere for at least 72 h without significant degradation [63,64].

**Figure 2:**
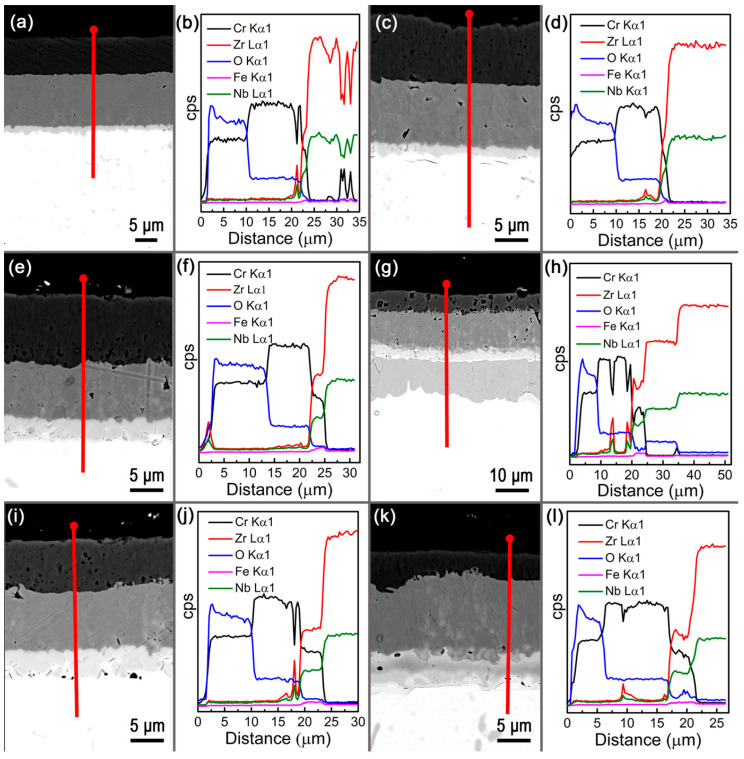
Cross-sectional SEM images and corresponding EDS line scans of the outer surface of the Cr-coated Zr tube oxidized at different conditions. (**a**,**b**) 1200 °C/0.5 h, (**c**,**d**) 1200 °C/1 h, (**e**,**f**) 1200 °C/2 h, (**g**,**h**) 1200 °C/4 h, (**i**,**j**) 1300 °C/0.5 h, (**k**,**l**) 1300 °C/1 h. Reproduced from [26] with permission from open access.

The main coating systems produced by laser cladding on zirconium substrates include:

- Chromium coatings (Cr, Cr-Al)—chromium coatings with thicknesses of 10–50 μm are currently the most advanced commercial solution. They are produced by laser cladding using Cr powder or CrAl wire [159,160]. Figure 2 shows Cross-sectional SEM images and corresponding EDS line scans of the outer surface of the Cr-coated Zr tube oxidized at different conditions. The main technical challenge is the elimination of microcracks at the Cr/Zr interface caused by differences in thermal expansion coefficients [161,162].
- Zr-Si and Zr-Si-Cr coatings—coatings containing silicon (5–15 wt%)—show exceptional oxidation resistance due to the formation of the tetragonal Zr-Si phase and a passive SiO_2_ layer [6,163]. The addition of chromium to the Zr-Si system further increases corrosion resistance [163].
- Composite coatings Zr-Al_2_O_3_ and Zr-MAX phases—the latest direction involves composite coatings containing hard ceramic phases Al_2_O_3_, SiC or MAX phases (e.g., Ti_3_SiC_2_, Cr_2_AlC) [164,165]. Zr-Al_2_O_3_ coatings exhibit hardness of 400–550 HV and wear resistance comparable to that of tool steels, while maintaining good substrate adhesion [166].

A key element in improving cladding properties is proper microstructure control through process parameter optimization. Studies show that increasing the cooling rate leads to grain refinement, which results in higher hardness and wear resistance, while excessively fast cooling may generate residual stresses and microcracks [167,168]. The introduction of intermediate transition layers with thermal expansion coefficients similar to the substrate, such as Ti-Al or Zr-Ni, reduces thermal mismatch and minimizes the risk of coating delamination [169].

The critical challenge of microcracking at Cr/Zr interfaces stems from the significant mismatch in coefficients of thermal expansion (CTE): chromium exhibits CTE values of approximately 11–12 × 10^−6^ K^−1^, whereas zirconium shows substantially lower values of 5–6 × 10^−6^ K^−1^. This mismatch generates interfacial stresses exceeding 300–400 MPa during thermal cycling, frequently leading to coating delamination [170]. Functionally graded materials (FGM) address this problem through compositional gradients that provide smooth thermomechanical property transitions, eliminating stress concentration at sharp interfaces [52]. Several advanced interlayer systems beyond conventional Ti-Al and Zr-Ni approaches have demonstrated superior performance. The Cr-CrN-Cr multilayer system utilizes chromium nitride as an intermediate layer with a CTE of approximately 10 × 10^−6^ K^−1^, serving as both a diffusion barrier and a thermal expansion buffer, reducing Cr-Zr interdiffusion by 65% at 1200 °C [5]. Silicide-based gradient systems (Zr → Zr_2_Si → ZrSi → Cr) leverage in situ formed Zr_2_Si barriers and protective SiO_2_ scales, achieving oxidation resistance two orders of magnitude higher than uncoated zirconium in 1200 °C steam [163]. MAX phase coatings based on Cr_2_AlC offer exceptionally high-temperature stability through Al_2_O_3_ scale formation, though rapid aluminum diffusion into zirconium requires additional diffusion barriers [165]. Composite systems with gradually increasing Al_2_O_3_ concentrations in the Zr-Cr matrix provide controlled hardness transitions (350→650 HV) with ceramic particles acting as crack deflection sites, improving thermal cycling resistance to over 500 cycles [166]. Optimized multilayer designs with five to seven discrete layers and CTE gradients limited to <2 × 10^−6^ K^−1^ between adjacent layers demonstrate potential to reduce interfacial thermal stresses by approximately 75% [171]. Laser cladding strategies for composition grading include dynamic powder mixing with dual feeders enabling real-time compositional control (±3% uniformity) [27], and post-deposition laser remelting at 50–70% of cladding power that reduces porosity from ~5% to <1% while promoting microstructural homogenization [172].

Advanced process monitoring and numerical modeling techniques are gaining increasing importance in the development of laser cladding technology. The use of vision systems, pyrometry and plasma spectroscopy enables real-time control of temperature and molten pool stability, while CFD and FEM modeling allow prediction of temperature, stress and material flow distributions [173,174]. Combining these tools with artificial intelligence and machine learning methods enables the development of adaptive control systems that automatically adjust process parameters in real-time, ensuring consistent coating quality [31,175].

## 6. Residual Stresses, Deformations and Cracking in Laser Processes of HCP Metals

The processes of laser welding and cladding of metals with a hexagonal close-packed (HCP) structure generate complex distributions of thermal and residual stresses resulting from extreme temperature gradients, differences in thermal expansion coefficients and a limited number of slip systems that enable strain relaxation. The high anisotropy of mechanical properties of HCP metals, combined with their low plasticity along the basal axis, makes laser joints in magnesium, titanium and zirconium particularly susceptible to microcracking and post-process deformation [6,47].

Residual stresses arise due to the nonuniform heating and cooling of the joint region, and their distribution depends on many factors: beam linear energy, weld geometry, travel speed and thermal properties of the material. In the fusion zone, tensile stresses typically dominate, whereas in the heat-affected zone compressive stresses form during solidification and cooling [176,177]. In titanium and its alloys, tensile components prevail along the weld axis, promoting microcrack initiation at defect concentration sites, especially in the transition region between the fusion zone and HAZ [178,179]. In magnesium, the stresses are more heterogeneous, resulting from low thermal conductivity and a large difference in expansion coefficients between the α-Mg and β-Mg_17_Al_12_ phases [180,181].

The mechanism of stress formation can be described as the result of local volumetric shrinkage mismatch during solidification and the nonuniform relaxation of plastic strains. The high cooling rate limits diffusion and leads to the retention of stresses within the weld microstructure. In HCP metals, where the number of slip systems is limited, stress relaxation is far less efficient than in FCC metals, which promotes microcrack and deformation formation [104,182]. In magnesium alloys, residual stresses often lead to local hot cracking, associated with the low solidus temperature and the presence of eutectic phases segregated at grain boundaries [183]. In titanium, transgranular microcracks are observed as a result of phase differences between the martensitic α′ zone and α + β dual-phase areas [184,185].

Residual stress analysis methods include both experimental and simulation techniques. The most commonly used are X-ray diffraction (XRD), neutron diffraction, Digital Image Correlation (DIC) and the hole-drilling method [186,187,188]. Measuring stresses in laser welds requires high spatial resolution due to the small width of the fusion zone. In titanium and magnesium, a strong dependence of stress distribution on crystallographic orientation and texture has been observed, confirmed by EBSD analysis and strain mapping [189,190]. Simulation methods, including finite element method (FEM) modeling, enable real-time reproduction of temperature and deformation fields as well as prediction of stress and strain evolution at various process stages [191,192].

Recent advances in coupled thermomechanical-microstructural modeling have enabled more accurate prediction of residual stresses in laser-welded HCP metals. For titanium alloys with a dual-phase α+β microstructure, finite element models incorporating solid-state phase transformation effects through the JMAK and Koistinen–Marburger equations demonstrate excellent correlation with experimental measurements [114,193]. These models integrate temperature-dependent thermodynamic parameters and phase transformation kinetics to quantitatively describe the relationship between microstructure evolution and residual stress generation [114,194].

Validation studies using X-ray diffraction, Digital Image Correlation, and neutron diffraction confirm that peak residual stresses in Ti-6Al-4V laser welds frequently occur in the heat-affected zone rather than the fusion zone center, with strong correlation to α′ martensite distribution and prior-β grain morphology [194,195]. For dual-phase titanium alloys, volumetric changes during β→α transformations induce transformation-induced plasticity effects that significantly modify final stress states [196]. Modern numerical models incorporating these phenomena achieve prediction accuracy within acceptable engineering tolerances when validated against experimental measurements [192,194,197].

For magnesium alloys, Crystal Plasticity FEM frameworks incorporating directional anisotropy have been developed [198,199], while zirconium models must additionally account for hydrogen diffusion and hydride precipitation effects [120,200,201]. The integration of thermodynamics-based phase transformations with elasto-plastic constitutive equations represents current state-of-the-art in predicting residual stresses across the complete thermal cycle [193,202].

Modern approaches to residual stress prediction increasingly employ multi-scale computational frameworks that integrate experimental measurements with numerical simulations. Digital Image Correlation (DIC) and Focused Ion Beam–DIC (FIB–DIC) provide spatially resolved strain fields [22,186,190] that serve as validation datasets for finite element models. These experimental-computational workflows form the basis for digital twin architectures, where real-time stress monitoring is coupled with predictive algorithms to enable adaptive process control. Such systems can dynamically adjust laser parameters based on predicted stress accumulation, preventing critical defect formation before it occurs.

Multi-technique experimental approaches combining X-ray diffraction, neutron diffraction, and Focused Ion Beam-Digital Image Correlation provide complementary validation datasets for residual stress models [22,186,190]. Recent validation studies demonstrate prediction accuracies within engineering tolerance when proper constitutive models and phase transformation kinetics are implemented [114,203]. For Ti-6Al-4V, correlation between predicted cooling rates and EBSD-observed microstructural features provides additional validation, as α′ martensite formation occurs predictably above critical cooling rates [2,46,114]. This multi-scale validation approach represents the best practice in contemporary modeling for HCP metals [204].

In numerical models, thermo-mechanical coupling is usually considered with temperature-dependent material properties. Studies show that the main contributor to stress generation is the temperature gradient through the weld thickness, responsible for bending moments and local deformations [205,206]. In laser cladding, it is observed that increasing beam travel speed reduces the width of the tensile stress zone, whereas too low a speed promotes stress concentration and crack development [207,208].

A key phenomenon affecting joint integrity is thermal cracking. In HCP metals, this phenomenon is particularly severe due to the limited ability for plastic deformation along the c-axis. In magnesium alloys, hot cracking occurs at temperatures between 500 and 600 °C, while in titanium it appears between 900 and 1100 °C [209,210]. These cracks usually develop along grain boundaries and in regions of secondary phase segregation, where local stresses exceed the material’s yield strength. In zirconium, despite its high ductility at elevated temperatures, cracking may be initiated by ZrH_x_ hydrides forming during cooling, which act as microcrack nuclei [200,201].

Effective mitigation of residual stresses and cracking in laser processes can be achieved using preventive methods such as preheating the material, controlled post-process cooling, multipass strategies, and energy distribution optimization [211,212,213]. In titanium and its alloys, spiral scanning and interlaced path strategies produce good results, ensuring uniform temperature distribution and minimizing stress concentration [197]. Additionally, active control using real-time modulation of power and scanning speed, based on optical and thermographic feedback, is increasingly applied in laser welding and cladding [214,215].

The use of post-processing techniques such as stress-relief annealing, Laser Shock Peening (LSP) or local surface remelting allows significant stress reduction and improved fatigue resistance for joints [216,217,218]. The LSP method, based on generating a shock wave through surface ablation, introduces beneficial compressive stresses in the near-surface layer, increasing the service life of titanium and zirconium components [219,220,221,222]. Recent studies also indicate the potential of ultrasonic and micro-vibration treatments to induce microscale plastic deformations compensating residual stresses [220,221,222,223,224,225].

In the context of laser cladding, ultrasonic vibration-assisted processing has demonstrated significant potential for defect mitigation through enhanced melt pool dynamics and improved microstructural homogeneity [135]. The application of ultrasonic assistance during laser processing of HCP metals promotes acoustic streaming effects that disrupt columnar grain growth, reduce porosity formation by 40–60% and decrease residual stress levels by 25–35% compared to conventional processing. This hybrid approach offers a promising strategy to address both residual stress accumulation and crack formation, particularly valuable for materials with limited slip systems such as magnesium and zirconium alloys.

The integration of in-process monitoring with post-processing optimization strategies represents a key capability of digital twin systems for HCP metals. By correlating real-time thermal signatures and acoustic emission signals with ex situ residual stress measurements [187,221], machine learning algorithms can predict the necessity and optimal parameters for post-weld treatments such as Laser Shock Peening or stress relief annealing. This predictive capability transforms reactive quality control into proactive process design, particularly valuable for HCP materials where stress-induced cracking can occur hours or days after processing due to delayed hydrogen embrittlement or phase transformations.

Comparative analysis of defect occurrence patterns (Table 3) reveals a clear progression in complexity and severity across HCP metals. Magnesium exhibits the highest porosity susceptibility due to low evaporation temperature (900 °C vs. 3287 °C for Ti), with pore frequencies 3–5 times higher than titanium under equivalent welding conditions [7,73,92]. Quantitatively, uncontrolled Mg welding produces 5–15% porosity, which can be reduced to <2% through combined application of beam oscillation (70–85% reduction) and vacuum processing (90–95% reduction) [7,16,73]. However, magnesium benefits from the absence of hydride-related defects and relatively low residual stress levels (80–150 MPa vs. 200–400 MPa in Ti), enabling simpler mitigation strategies focused primarily on vapor pressure management [180,181]. Titanium presents intermediate defect complexity dominated by phase transformation issues α’ martensite formation occurs in 85–95% of laser welds without controlled cooling, causing hardness increases of 100–150 HV but reducing ductility by 30–50% [67,110,184]. The most effective mitigation combines controlled cooling rates (50–200 K/s achieving 70–85% crack reduction) with post-weld heat treatment at 700–800 °C (80–95% crack elimination and 80–90% microstructural homogenization) [114,115,117]. The moderate gas absorption sensitivity (tolerable impurity levels 50–100 ppm vs. <20 ppm for Zr) allows industrial implementation with high-purity shielding rather than hermetic chambers [2,19,50]. Zirconium exhibits the most critical defect profile, where hydride formation above 50 ppm hydrogen causes catastrophic embrittlement, reducing fracture toughness by 60–80% and necessitating ultra-clean atmosphere control (<10 ppm H_2_, <20 ppm O_2_/N_2_) unavailable for Mg or Ti processing [68,120,201]. The effectiveness hierarchy for Zr is stark: atmosphere control below 10 ppm H_2_O achieves 90–95% hydride reduction, pre-weld degassing at 400 °C for 2 h provides 80–90% reduction, while ultra-clean processing (<5 ppm H_2_) enables 95–99% elimination [50,52,53,54]. Quantitatively, elimination method complexity scales proportionally: magnesium defects require 2–3 simultaneous control measures achieving 70–90% defect reduction, titanium requires 4–5 measures achieving 75–95% reduction, while zirconium demands 6–8 stringent controls achieving 90–99% reduction but at 3–5 higher cost [50,51,52,53,54,211,212,213]. This progression directly correlates with the 1:2:5 relative cost ratio and explains the technology readiness level differences (TRL 7–8 for Mg, 8–9 for Ti, 6–7 for Zr).

## 7. Numerical Modeling and Simulation of Laser Processes in HCP Metals

Numerical modeling and computer simulations constitute an indispensable component of contemporary research on laser welding, cladding and remelting processes of metals with a hexagonal close-packed (HCP) structure. The complex nature of these phenomena, including simultaneous heat conduction, fluid flow, evaporation, solidification and stress development, makes direct observation and real-time control of parameters difficult to achieve. For this reason, mathematical and numerical models are used to analyze temperature distributions, flow velocities, thermal stresses, pore formation and the dynamics of the keyhole channel.

The first models describing laser welding processes were semi-analytical and based on the unsteady-state Fourier equation with the simplified assumption of a point heat source. Modern models now include full thermo-hydrodynamic coupling and account for phase transformations and surface evaporation. In laser processing of HCP metals, thermal conductivity anisotropy plays a particularly important role, as it affects the temperature distribution and the shape of the molten metal pool [76,225]. In titanium and zirconium alloys, these phenomena are further modified by the temperature-dependent variability of material properties, especially the abrupt changes in specific heat and thermal expansion coefficient during phase transformations [226].

One of the most commonly used simulation tools is the finite element method (FEM), which enables numerical solutions of heat conduction and Navier–Stokes equations describing molten metal flow. These models also incorporate Marangoni forces and vapor pressure effects in the keyhole channel, allowing reproduction of weld shape and prediction of surface instabilities [227,228]. In advanced Computational Fluid Dynamics (CFD) analyses, two-phase (gas–liquid) modeling is also applied, describing mass and momentum transfer between metal vapor and the liquid pool. This allows simulation of phenomena such as keyhole pulsation, collapse and pore formation [193,229].

CFD and FEM simulations are often integrated into coupled thermo-mechanical–fluid models that allow simultaneous tracking of stress and strain evolution during metal solidification. In titanium alloys, such models enable the prediction of local stress concentrations in regions with varying microstructures, which is critical for assessing hot-cracking risk [230,231,232]. In magnesium alloys, crystallization models incorporating grain orientation and dendrite growth direction are used to predict joint texture and secondary phase morphology [233,234].

Latest predictive models combine physics-based FEM with machine learning for real-time residual stress prediction [235,236]. Physics-informed neural networks trained on FEM datasets achieve high prediction accuracy while reducing computational time by orders of magnitude [235,237]. For HCP metals, hybrid approaches integrating thermodynamic databases, kinetic models, and data-driven algorithms provide robust predictions [238,239]. Recent applications to Ti-6Al-4V successfully predict both residual stress distribution and microstructural features when validated experimentally [32,203,236]. Integration of in situ monitoring enables closed-loop control systems for minimizing stress accumulation and cracking [240,241].

Recent advancements in numerical methods have introduced multiscale coupled modeling approaches. In this type of framework, macroscopic simulations (temperature distributions and heat flow) are combined with microscopic models (crystallization, phase transformations, microstructure evolution). This enables prediction of the local joint microstructure based on real thermal and hydrodynamic conditions [127,242,243]. Models based on the Phase Field Method (PFM) allow for the description of grain boundary evolution and dendrite growth in real-time, which is particularly useful for the analysis of titanium and zirconium alloys [244,245,246].

In stress and deformation modeling, elasto-plastic models that account for temperature-dependent material properties and the effects of phase transformations play a major role. In titanium alloys, such models employ constitutive equations describing the behavior of α, β and α′ phases, which allows prediction of their volumetric fractions and influence on stress distribution [247,248]. In magnesium alloys, Crystal Plasticity FEM (CPFEM) models are used, incorporating directional deformation anisotropy and a limited number of slip systems [198,199].

A significant development direction involves the use of machine learning and artificial intelligence in modeling laser processes. Algorithms based on neural networks, deep learning and Support Vector Machines (SVM) are employed to predict weld shape, temperature distribution, pore formation and stress evolution based on process data [31,237]. The integration of physical models with machine learning systems leads to the creation of hybrid models that can autonomously correct prediction errors based on real-time measurement data [240,249]. In many cases, such solutions reduce simulation time by as much as 60–70% while maintaining high prediction accuracy [250].

Machine learning models for HCP metal laser processing utilize material-specific input features and prediction targets addressing unique physical phenomena. Input features comprise three categories: (1) process parameters—laser power (1.5–6 kW), scanning speed (0.3–6 m/min), focal position (±2 mm), beam diameter (0.2–0.9 mm), gas flow rate (10–50 L/min), plus derived parameters (linear energy, power density, interaction time) [2,7,16,19,55]; (2) thermal monitoring—peak temperature (650–1100 °C Mg, 1650–2000 °C Ti, 1850–2100 °C Zr), thermal gradients (10^4^–10^6^ K/m), cooling rate (10^2^–10^5^ K/s) from thermography at 2–10 kHz [13,80,101]; (3) optical/acoustic signals—keyhole depth, aspect ratio (stable processing: 3.5–4.5 Mg, 4.0–6.0 Ti, 3.0–4.5 Zr), depth fluctuation (<15% stable), plasma intensity and acoustic emission (1–100 kHz) [11,12,13,31,98,173]. Output predictions address material-specific challenges: porosity fraction (0–15%, target accuracy ±0.5–1%) for magnesium [13,31]; α’ martensite content (0–100%, ±5%) and hardness (300–450 HV, ±10–15 HV) for titanium [32,67,111]; and hydride risk (binary classification, >95% accuracy) and oxide thickness (5–100 µm, ±5 µm) for zirconium [25,50,57,122]. Mechanical properties including residual stress (±30–50 MPa accuracy) and tensile strength (±3%) represent common targets across all HCP metals [22,178,179,180,181].

Magnesium—Porosity Prediction:

Deep belief networks (DBN) with 45 input features (process parameters, thermal signatures, keyhole morphology, plasma spectroscopy) predict porosity with MAE = 0.6%, R^2^ = 0.89 on 2847 weld samples [13,31]. Top predictive features: keyhole depth fluctuation (importance 0.24), peak temperature (0.19) and linear energy (0.15). Binary classification (defect-free < 1% vs. defective > 3% porosity) achieves 92% accuracy [13,31,237]. Real-time CNN implementation processing coaxial NIR and blue-light images achieves 87% accuracy at 50 Hz, enabling closed-loop control with <100 ms response [13,31].

Titanium—Microstructure and Martensite:

Deep neural networks predict α’ martensite fraction using 28 features (process parameters, thermal history including at 700–1000 °C intervals, β-transus crossing dynamics) with MAE = 4.2%, R^2^ = 0.94 on 3452 Ti-6Al-4V samples [32,67,111]. Critical features: cooling rate at 800 °C (importance 0.31), peak temperature (0.22) and linear energy (0.18). Hardness prediction achieves MAE = 12 HV, R^2^ = 0.91; tensile strength MAE = 28 MPa, R^2^ = 0.88 [32,109]. CNN analysis of surface topography (8 µm resolution) classifies microstructure (fully martensitic >95% α’, mixed 40–95%, lamellar <40%) with 96% accuracy [32]. Physics-informed neural networks (PINN) incorporating heat diffusion and phase transformation equations reduce training data requirements by 60% while maintaining R^2^ = 0.92 for martensite prediction [236,237].

Zirconium—Hydride Formation and Contamination:

Support vector machines (SVM) with 22 features including plasma H-alpha (656.3 nm) and H-beta (486.1 nm) line intensities plus atmosphere monitoring (O_2_, N_2_, H_2_ partial pressures) achieve 97% accuracy for hydride risk classification (safe <50 ppm H_2_ vs. critical >50 ppm), with precision 96%, recall 98% on 1247 welds [25,50,57]. Random forest regression predicts oxide thickness with MAE = 4.2 µm, R^2^ = 0.87 using 18 features [55,122]. For ATF coating applications, gradient boosting machines integrating multi-sensor data predict coating quality (thickness uniformity ±10 µm, dilution percentage 5–15%, adhesion strength > 50 MPa) with 93% classification accuracy [27,160].

Comparative performance correlates with process window width: magnesium models achieve 92% defect prediction accuracy due to dominant single-mechanism (evaporation porosity); titanium models demonstrate R^2^ = 0.94 for microstructure but 88–91% overall defect detection due to complex interactions (porosity, cracking, gas absorption); and zirconium models show 97% binary classification for critical hydride risk but lower continuous regression (R^2^ = 0.85–0.87) due to limited datasets [13,25,31,32,57,122]. Inference latency varies: lightweight CNN 20–50 ms (20–50 Hz), DNN 50–100 ms (10–20 Hz), ensemble methods 100–200 ms (5–10 Hz) [13,32,237,240].

The integration of physics-based models with machine learning approaches discussed in this section represents the foundational architecture for digital twin systems. Unlike standalone simulations, digital twins maintain bidirectional communication between physical processes and their computational representations, continuously updating model parameters based on sensor feedback. For HCP metals, this architecture is particularly valuable due to the sensitivity of process outcomes to minor parameter variations. For example, keyhole stability monitoring data (Section 3) can be fed in real-time to CFD models to predict porosity formation, while residual stress measurements (Section 6) validate thermo-mechanical FEM predictions and trigger corrective actions. The concept of digital twins extends beyond process monitoring to encompass the entire component lifecycle, from design through manufacturing to in-service performance, as detailed in Section 8.

For titanium alloys, machine learning models predict α’ martensite fraction and hardness distribution using input parameters encompassing thermal history data (cooling rates of 10^3^–10^5^ K/s), process parameters (power, speed, beam diameter) and in situ temperature measurements. Advanced multi-sensor fusion approaches integrating coaxial cameras, pyrometry and plasma spectroscopy have demonstrated real-time prediction of microstructural features and defect formation in titanium alloys with accuracy exceeding 95%, representing a significant advancement in intelligent process control [135]. Deep learning approaches utilizing surface topography images captured by high-resolution cameras (spatial resolution <10 µm) successfully correlate process parameters with α’ martensite content (0–100%) and hardness values (300–450 HV) in Ti-6Al-4V laser welds and additively manufactured components [32,109,111].

**Figure 3:**
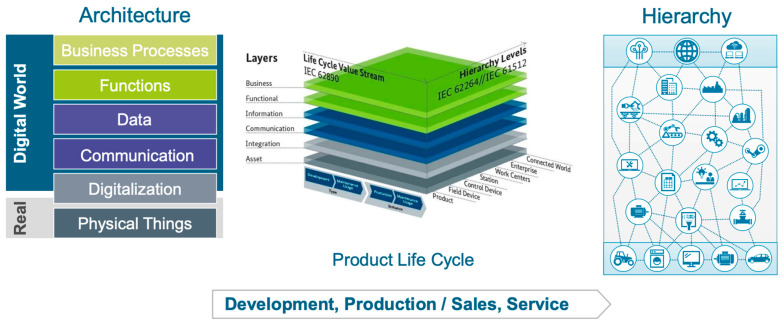
Reference Architecture of Industry 4.0 (RAMI 4.0) model as a solution space with a coordinate system for applications in advanced metal manufacturing processes. Reproduced from [251] with permission from open access.

Simulations coupled with experimental data now form the basis for building digital twins of laser processes. These models reproduce the entire manufacturing cycle from laser source parameters to the resulting microstructure and joint properties enabling full optimization and real-time quality control. Integrating advanced laser technologies into Industry 4.0 requires the use of structured reference models such as RAMI 4.0 (Reference Architectural Model Industrie 4.0), which defines a three-dimensional space encompassing the product lifecycle, the production system hierarchy, and the architectural layers from physical components to business processes (Figure 3).

The digital twin concept is particularly promising in the context of HCP metallurgy, where even small deviations in process parameters can lead to significant changes in microstructure and residual stresses.

## 8. Summary and Development Perspectives of Laser Technologies for HCP Metals

The dynamic development of laser technologies in recent decades has significantly expanded the possibilities for processing metals with a hexagonal close-packed (HCP) structure, such as magnesium, titanium and zirconium. Due to precise energy control, high power density and a minimal heat-affected zone, laser processes have become a key tool in the welding, cladding and regeneration of these materials, which have traditionally been considered difficult to weld. The studies presented in the previous chapters demonstrate that the use of modern laser systems supported by numerical analysis and adaptive control enables the production of joints and coatings of very high quality and operational stability [104,252]. Table 4 presents main defects in laser processing of HCP metals and methods of their elimination. The qualitative process window characterizations in Table 4 (“moderate” for Mg, “narrow” for Ti, “very narrow” for Zr) reflect quantifiable mechanistic relationships with the fundamental physicochemical properties presented in Table 1. Understanding these correlations is essential for rational process design and parameter optimization.

The fundamental physicochemical properties presented in Table 1 directly determine the process window width for each HCP metal through several quantifiable mechanistic relationships. This correlation can be understood through systematic analysis of key property-parameter linkages.

Magnesium Process Window Analysis:

Magnesium exhibits a moderate process window primarily due to the combination of three favorable properties from Table 1: (1) wide temperature range between melting (650 °C) and intensive vaporization onset (~900 °C), providing a 250 °C thermal buffer zone for process control [7,35]; (2) high thermal conductivity (156 W/m·K, 7-fold higher than Ti) enabling effective heat dissipation and reduced thermal gradient severity [35]; and (3) moderate vapor pressure (10^5^ Pa at melting temperature) requiring careful but achievable evaporation management [1,7]. These properties translate to approximately 8–15 viable parameter combinations within laser power ranges of 1.5–4 kW and welding speeds of 1–6 m/min [7,16]. The linear energy tolerance of ±50% (80–400 J/mm) allows significant operational flexibility [7,25]. However, the process is constrained by high vapor pressure necessitating careful power modulation and shielding gas optimization to prevent porosity formation, which occurs in 5–15% of uncontrolled welds [7,73].

Titanium Process Window Analysis:

Titanium presents a narrow process window due to the unfavorable combination of: (1) high melting temperature (1668 °C, 2.6× higher than Mg) requiring laser powers of 3–5 kW [2,19,21]; (2) very low thermal conductivity (21.9 W/m·K, 87% lower than Mg) causing steep thermal gradients (∇T > 10^6^ K/m) and significant residual stress accumulation (200–400 MPa vs. 80–150 MPa in Mg) [2,33,178,179]; and (3) extreme sensitivity to atmospheric contamination where even 100–200 ppm of oxygen or nitrogen causes embrittlement through α’ martensite formation and gas absorption [2,19,45]. The rapid α/β phase transformations during cooling (10^3^–10^5^ K/s, Table 1) further restrict the acceptable parameter range, as deviations of ±20% in cooling rate alter mechanical properties by 30–40% through changes in martensitic content [46,67]. This results in only 4–6 viable parameter combinations with ±30% linear energy tolerance (150–280 J/mm) [2,21]. The peak residual stresses of 200–400 MPa [178,179] arise from the combination of low thermal conductivity preventing stress dissipation, limited slip systems in HCP α-phase restricting plastic relaxation, and volumetric changes during β→α transformation [33,184,185].

Zirconium Process Window Analysis:

Zirconium exhibits the narrowest process window due to simultaneous occurrence of the most extreme property combinations from Table 1: (1) highest melting temperature (1855 °C, 11% higher than Ti) requiring 2.5–4.5 kW laser power but with minimal thermal margin [3,55,56]; (2) lowest tolerance for contamination (<20 ppm O_2_/N_2_/H_2_ compared to 100–200 ppm for Ti) driven by catastrophic hydride formation at hydrogen levels above 50 ppm, which reduces fracture toughness by 60–80% [3,50,68,120]; (3) extremely low vapor pressure at melting (10^−1^ Pa, 10^6^-fold lower than Mg) theoretically favorable but offset by hypersensitivity to trace impurities [3,49]; and (4) narrow thermal expansion coefficient (5.7 × 10^−6^ K^−1^, 78% lower than Mg) which, combined with limited HCP slip systems, prevents effective stress relaxation leading to 150–300 MPa residual stresses [49,122,201]. The linear energy window for defect-free Zr welding is restricted to 100–200 J/mm with only ±10% tolerance [25,51,55] compared to 80–400 J/mm with ±50% tolerance for magnesium. This translates to merely 2–3 acceptable parameter combinations requiring hermetic chamber processing with continuous atmosphere monitoring systems (capital cost USD500k-2M per installation) [51,52,53,54].

Quantitative Property-Process Correlations:

These mechanistic relationships explain the observed hierarchy in processing difficulty. The required atmosphere purity scales exponentially with reactivity: Mg tolerates <100 ppm impurities (standard industrial gas protection, USD 20k–80k equipment cost), Ti requires <50 ppm (high-purity shielding, USD50k–200k), while Zr demands <20 ppm O_2_/N_2_ and <10 ppm H_2_ (hermetic chambers, USD500k–2M) [2,39,50,51,52,53,54]. The defect formation sensitivity follows similar progression: Mg porosity reduces 70–90% with 2–3 control measures, Ti cracking reduces 75–95% with 4–5 measures, while Zr hydride prevention requires 6–8 stringent controls achieving 90–99% elimination [50,51,52,53,54,211,212,213]. Process speed decreases inversely with melting temperature: Mg (40–60 mm/s) → Ti (15–30 mm/s) → Zr (10–20 mm/s), directly impacting productivity and cost [16,19,39,55]. The 1:1.5–2.0:3.0–5.0 relative cost ratio (Table 4) reflects these compounding factors: equipment sophistication, reduced throughput and intensive quality control requirements [1,2,3,50,51,52].

Industrial Implications:

These quantitative property-process-performance relationships explain industrial adoption patterns. Titanium dominates high-value aerospace and biomedical applications (TRL 8–9) where 200–400% cost premium over Mg is justified by superior strength-to-weight ratio and biocompatibility [2,43,252]. Magnesium finds niche automotive and electronics applications (TRL 7–8) balancing lightweight performance with moderate processing costs [1,35,252]. Zirconium remains largely confined to nuclear applications (TRL 6–7) where extreme reliability requirements (low neutron absorption cross-section, high-temperature corrosion resistance) justify prohibitive 300–500% cost premium over Ti and extensive processing infrastructure [3,63,64]. The TRL progression inversely correlates with process window width, confirming that broader parameter tolerance accelerates industrial maturity.

In the case of magnesium alloys, precise control of process parameters and the use of protective atmospheres that limit oxidation and metal evaporation are of key importance. The introduction of alloying additions, composite powders and laser cladding technologies enables a significant increase in corrosion and tribological resistance. Titanium and its alloys, widely used in the aerospace and biomedical industries, represent a material in which the development of laser technologies has brought the greatest progress in joint quality and microstructure control precision. High atmospheric purity, the use of multitrack scanning and hybrid techniques (laser–arc, laser–plasma) make it possible to obtain welds with minimal defects and low residual stresses [253,254,255]. In zirconium, whose applications are mainly concentrated in nuclear energy, laser processing allows the production of protective coatings with improved resistance to oxidation, hydride formation and corrosion in extreme environments [256,257].

A key factor determining the success of laser processes in HCP materials is the understanding and control of the microstructure formed during rapid solidification. Due to the intensive development of characterization techniques such as scanning and transmission electron microscopy (SEM, TEM), electron backscatter diffraction (EBSD) and X-ray spectroscopy, it is now possible to precisely track grain morphology, crystallographic orientation and phase transformations within the joints [258,259,260]. Modern microstructural studies combined with computational methods make it possible to identify correlations between process parameters, weld geometry and mechanical properties of joints, providing a foundation for further optimization of laser technologies [261,262].

The integration of numerical modeling and machine learning systems plays an increasingly important role in the advancement of laser technologies. Thermomechanical models and CFD–FEM simulations coupled with phase-field methods enable real-time prediction of microstructure, residual stresses and defect formation. The use of artificial intelligence allows process data analysis and automatic adjustment of parameters to maintain melt pool stability and joint quality [262,263,264,265]. Such solutions are leading to the development of so-called intelligent laser welding and cladding systems, in which the process is adaptively controlled based on sensor data and predictions of thermal and mechanical changes.

One of the most important development directions is the concept of the digital twin, which integrates sensor data, physical models, numerical analyses and machine learning algorithms into a single predictive system. This approach enables real-time monitoring, analysis and correction of processes, significantly increasing repeatability and manufacturing efficiency. Digital twins of laser processes for HCP metals allow prediction not only of weld geometry but also of microstructure and stress distribution, forming the foundation for the concept of self-optimizing manufacturing [266,267].

The digital twin framework for HCP metal laser processing synthesizes the knowledge presented throughout this review. Physical mechanisms governing keyhole dynamics, melt pool convection and solidification (Section 3) provide the fundamental equations for real-time simulations. Microstructural models predicting phase transformations and grain morphology (Section 4) enable property forecasting. Residual stress prediction algorithms (Section 6) prevent defect formation through adaptive parameter control. Numerical modeling techniques (Section 7) form the computational backbone, while machine learning algorithms trained on historical process data enable rapid decision-making. This hierarchical integration transforms isolated research findings into actionable industrial tools, addressing the specific challenges of HCP materials: limited process windows, sensitivity to atmospheric contamination and propensity for stress-induced cracking.

The digital twin concept for manufacturing processes is currently being standardized within international standards, in particular ISO 23247, which defines the reference architecture, functional requirements and data exchange protocols for digital twins of manufacturing systems, and ISO 23704-3, which specifies the principles for building cyber-physical control systems in an industrial environment. Implementation of these standards in the coming years will enable full interoperability of digital twin systems in global supply chains and ensure consistency in the real-time monitoring, prediction and optimization of HCP metal laser processes [268,269].

In the context of industrial applications, further development of laser technologies focuses on improving energy efficiency and process productivity. This includes the implementation of high-power lasers with wavelengths adapted to material absorption, the use of ultrashort-pulse sources for precision micromachining and the integration of additive technologies (DED, L-PBF) with repair and regenerative processes [10,270,271,272,273]. Another important trend is the development of functional coatings deposited by laser cladding and remelting methods, enabling surfaces to gain specific properties—from wear resistance to bioactivity and self-cleaning capabilities [274].

The industrial scalability and economic viability of laser technologies for HCP metals are strongly differentiated by metal type, with barriers ranging from moderate (Mg) to severe (Zr). For magnesium alloys, the primary economic advantages include relatively low infrastructure costs—standard shielding gas systems with argon or argon-helium mixtures (purity 99.9%, cost ~USD 2–5/m^3^) are sufficient, and welding can be performed in open atmosphere with local gas protection [7,39]. The high process speed (1–6 m/min) enables productivity comparable to conventional arc welding, with laser welding cycle times typically 40–60% shorter than GMAW for thin-walled components [16,17]. However, the limited industrial adoption of magnesium alloys restricts market scale, resulting in higher per-part laser processing costs due to smaller production volumes and limited specialized equipment availability. Powder recycling in laser cladding of Mg alloys presents moderate challenges, with reuse rates of 70–85% achievable through sieving (particle size 45–150 μm) and moisture control, though oxidation during storage remains problematic [136,138].

For titanium alloys, the economic barriers are substantially higher due to stringent atmospheric requirements. High-purity argon or helium shielding (purity > 99.99%, cost USD 15–40/m^3^ for helium) increases operating costs by 300–500% compared to standard welding gases [2,45]. Localized shielding systems for Ti-6Al-4V laser welding consume 20–50 L/min of high-purity gas, translating to USD5–15 per meter of weld [19,21]. The moderate process speeds (0.5–2 m/min) and narrow process window (requiring extensive parameter development and quality control) further increase per-part costs by an estimated 200–400% compared to magnesium. However, the large-scale industrial applications in aerospace and biomedical sectors (global Ti welding market >USD2 billion annually) enable cost amortization through high production volumes [43,252]. In laser cladding and additive manufacturing, powder recycling efficiency reaches 85–95% for Ti alloys through controlled atmosphere handling and particle size distribution maintenance, though powder degradation after 5–8 reuse cycles (oxygen pickup > 0.35 wt%) necessitates fresh powder supplementation at 10–15% per cycle [23,133,254].

Zirconium alloys present the most severe economic and scalability barriers, primarily due to ultra-clean atmosphere requirements. Hermetically sealed welding chambers with continuous atmospheric monitoring systems (maintaining <10 ppm O_2_/N_2_/H_2_) require capital investments of USD500,000–2,000,000 per installation, compared to USD50,000–200,000 for Ti welding systems and USD20,000–80,000 for Mg systems [51,53]. Helium recirculation and purification systems (purity 99.99%, <5 ppm impurities) consume 5–15 m^3^/h, with operational costs of USD200–600 per hour excluding labor and depreciation [54]. The chamber preparation cycle (evacuation, purging, atmosphere stabilization) requires 2–4 h before welding can commence, severely limiting throughput for small-batch production typical in nuclear applications [52,57]. The extremely low process speeds (0.3–0.8 m/min) combined with the narrow process window (only 2–3 viable parameter combinations) result in welding cycle times 5–8 times longer than for titanium and 8–12 times longer than for magnesium, with estimated per-meter costs of USD150–400 compared to USD20–60 for Ti and USD5–15 for Mg [3,25,51]. Powder recycling in Zr laser cladding for ATF coatings is particularly challenging, as contamination tolerances of <50 ppm for oxygen and hydrogen necessitate handling in gloveboxes with <1 ppm atmosphere purity, limiting practical reuse to 2–3 cycles with recovery rates of only 60–75% [27,52]. These economic barriers restrict laser processing of zirconium to high-value nuclear applications where performance requirements justify costs, preventing broader industrial adoption despite technical feasibility.

A major challenge remaining is the full control over the formation of defects such as porosity, cracking and element segregation, which can limit the durability and reliability of joints. The development of hybrid joining methods combining lasers with additional sources of heat or mechanical energy opens new possibilities for effectively mitigating these issues. Progress in this area requires close collaboration between research centers and industry, as well as the development of open material and process databases supporting the design of new alloys dedicated to laser technologies [273].

Laser technologies in the processing of metals with an HCP structure are currently undergoing dynamic development and represent a key component of modern engineering. The integration of experimental, simulation and computational research with artificial intelligence tools is leading to the emergence of a new generation of laser processes intelligent, self-adaptive and fully controlled. Future research will focus on developing comprehensive monitoring and control strategies that will further improve the quality, efficiency and reliability of joints in magnesium, titanium and zirconium materials.

## Figures and Tables

**Table 1:** Comparison of physicochemical and welding properties of HCP metals.

Property	Magnesium (Mg)	Titanium (Ti)	Zirconium (Zr)
Crystal structure	HCP	HCP (α), BCC (β)	HCP (α), BCC (β), HCP (ω) [61]
Density [g/cm^3^]	1.74 [35]	4.51 [33,43]	6.52 [49]
Melting point [°C]	650 [35]	1668 [33]	1855 [3,49,62]
Boiling point [°C]	1090 [1]	3287 [43]	4409 [49]
Thermal conductivity [W/m·K]	156 [35]	21.9 [33]	22.7 [3]
Thermal expansion coefficient [10^−6^/K]	25–27 [35]	8.6 [33,44]	5.7 [49]
Vapor pressure at melting temperature [Pa]	~10^5^ [1,7]	~10^2^ [2]	~10^−1^ [3]
Main welding issues	Evaporation, oxidation, porosity, hot cracking [1,37,38]	Gas absorption, α′ martensite, residual stresses [2,6,45]	Hydride formation, oxidation, strict atmosphere purity requirements [3,25,49]
Typical shielding atmosphere	Ar, He, Ar + He, vaccum [16,39]	Ar, He, vaccum [2,45]	High-purity He, vacuum (<20 ppm O_2_, N_2_, H_2_) [3,50,51,52]
Minimum atmosphere purity [ppm]	<100 [39]	<50 [2,19]	<20 [52,54]
Main applications	Automotive, aerospace, electronics [35,36]	Aerospace, medical, chemical industry [43,44]	Nuclear energy, chemical industry [3,49,63,64,65,66]

**Table 2:** Typical laser welding parameters of HCP metals.

Parameter	Magnesium (AZ31, AZ91)	Titanium (Ti-6Al-4V)	Zirconium (Zr-2, Zr-4)
Laser type	Nd:YAG, fiber, green (532 nm) [16,87,88,89]	Nd:YAG, fiber, disk [2,10]	Fiber, Nd:YAG [55,56]
Beam power [kW]	1.5–4.0 [7,17]	2.0–6.0 [2,19,21]	2.5–5.0 [55,122]
Welding speed [mm/s]	30–80 [16,39]	10–40 [19,20,48]	8–25 [55,57]
Linear energy [J/mm]	20–80 [7,40]	100–400 [2,21]	150–500 [55,122]
Focal spot diameter [mm]	0.2–0.6 [17]	0.3–0.8 [19,21]	0.4–0.9 [55]
Power density [MW/cm^2^]	0.8–2.5 [10,13]	1.5–4.0 [10,12]	1.2–3.5 [55]
Focal position [mm]	0 do +2 (above surface) [17,89]	−1 do +1 [21]	−2 do 0 [55]
Shielding gas flow rate [L/min]	15–30 [16,39]	20–40 [2,19]	25–50 [52,54]
Vacuum chamber	Optional [7]	Recommended for high quality [2,45]	Mandatory (*p* < 10^−2^ mbar) [3,52]
Operating mode	Continuous Wave (CW), modulated [16,17]	Continuous Wave (CW), pulsed [2,19]	Continuous Wave (CW) [55,57]
Penetration depth [mm]	1.5–4.0 [7,39]	2.0–8.0 [2,48]	1.5–5.0 [55,122]
Weld width [mm]	2.0–5.0 [39,40]	1.5–4.5 [19,21]	1.8–4.0 [55]
Cooling rate [K/s]	10^3^–10^4^ [39,80]	10^3^–10^5^ [67,80]	10^2^–10^4^ [122]
Weld hardness [HV]	60–85 [39]	350–450 [67,122]	220–280 [122,123]

**Table 3:** Main defects in laser processing of HCP metals and methods of their elimination.

Defect Type	Magnesium	Titanium	Zirconium	Elimination Methods	Quantitative Effectiveness
Porosity	Very frequent (Mg evaporation, 5–15% without control) [7,73]	Moderate (dissolved gases, 2–8%) [92,95]	Rare (under pure atmosphere, <1%) [52]	Power reduction [12,14]. Beam modulation [16,17]. Beam oscillation [73,74]. Vacuum/low pressure [7,16]. Optimization of welding speed [13,15]	Power modulation: 60–80% porosity reduction in Mg [16,17]. Beam oscillation: 70–85% reduction in Ti [73,74]. Vacuum processing: 90–95% reduction in Mg [7]. Speed optimization: 40–60% reduction across HCP metals [13,15]
Hot cracking	Frequent (low solidus temperature, crack density 5–20 mm/m) [37,183]	Rare (<1 mm/m) [184]	Very rare (<0.5 mm/m) [122]	Preheating [211,212,213]. Control of linear energy [16,40]. Multipass welding [107]. Alloying additions [106]	Preheating (200–250 °C): 75–90% crack reduction in Mg [211]. Linear energy control: 50–70% reduction in Mg [16,40]. Multipass welding: 60–80% reduction in Ti [107]. Alloying (Al, Zn): 85–95% crack elimination in Mg [106]
Cold cracking	Rare	Moderate (α′ martensite, 2–8 mm/m) [67,184,185]	Possible (hydrides, 1–5 mm/m) [200,201]	Stress-relief annealing [114,115,211]. Cooling rate control [117]. Optimization of welding sequence [197,209]	Annealing (700–800 °C): 80–95% crack elimination in Ti [113,114]. Cooling rate control (50–200 K/s): 70–85% reduction in Ti [117] H_2_ control (<10 ppm): 90–98% reduction in Zr [50,51,52]
Oxidation/nitriding	Very intense (oxide 50–200 µm) [35,37]	Intense (10–50 µm) [43,45]	Critical (5–30 µm, embrittlement) [49,50]	Vacuum chamber [7,52]. High-purity He/Ar [2,39,52]. Additional gas shielding [19]. Ceramic backing plates [2]	Vacuum (<10^−2^ mbar): 95–99% reduction [7,52]. High-purity gas (99.999%): 85–95% reduction in Ti [2,45]. Shielding optimization: 60–80% reduction in Mg [39]
Hydride formation	Not observed	Possible (100–500 ppm H_2_ absorption) [95]	Highly critical (>50 ppm causes embrittlement) [68,120,200,201]	Minimization of H_2_O in atmosphere [52,54]. Material degassing [53]. Chamber purity < 20 ppm H_2_ [50,52]	Atmosphere control (<10 ppm H_2_O): 90–95% reduction in Zr [52,54]. Pre-weld degassing (400 °C, 2 h): 80–90% reduction in Zr [53]. Ultra-clean (<5 ppm H_2_): 95–99% elimination in Zr [50]
Nonuniform microstructure	Frequent (β-Mg_17_Al_12_ segregation, hardness variation 15–25 HV) [39,106]	Frequent (α′ and α + β, hardness variation 50–100 HV) [67,109,110]	Moderate (hardness variation 20–40 HV) [118,119]	Cooling rate control [80,117]. Post-weld heat treatment [114,115]. Multitrack scanning [197,215]	Controlled cooling: 50–70% uniformity improvement [80,117] PWHT (700 °C, 2 h): 80–90% homogenization in Ti [114,115]. Optimized scanning: 60–75% improvement [197,215]
Residual stresses	Moderate (80–150 MPa tensile) [180,181]	High (200–400 MPa tensile) [178,179,184]	High (150–300 MPa tensile) [122,201]	LSP (Laser Shock Peening) [216,217,218,219,220,221,222] Stress-relief annealing [211,212,213]. Sequence control [197,209]. Preheating [211]	LSP: 40–60% reduction, introduces 100–200 MPa compressive [216,217,218,219,220,221,222]. Annealing: 60–80% stress relief [211,212,213]. Sequence optimization: 30–50% reduction [197,209]. Preheating (200–400 °C): 40–60% reduction [211]
Low fatigue strength	Frequent (50–70% of base metal) [39]	Possible (60–80% of base metal) [113,185]	Rare (85–95% of base metal) [122]	Pore elimination [12,14,74] LSP [216,217,218,219,220,221,222]. Microstructure control [108,117]	Combined defect control: 80–95% fatigue recovery in Mg [12,39] LSP: 150–300% improvement in Ti [216,217,218,219,220,221,222]. Microstructure optimization: 100–150% improvement in Ti [108,117]

**Table 4:** The qualitative process window characterizations of HCP metals.

Aspect	Magnesium	Titanium	Zirconium	Mechanistic Correlation to Table 1
Optimal laser power [kW]	2.0–3.5 [7,16,17]	3.0–5.0 [2,19,21]	2.5–4.5 [55,56]	Scales with melting point (650 °C → 1668 °C → 1855 °C) and inversely with thermal conductivity (156 → 21.9 → 22.7 W/m·K)
Optimal welding speed [mm/s]	40–60 [16,39,40]	15–30 [19,20]	10–20 [55,57]	Inversely correlates with melting temperature; governed by keyhole stability time
Linear energy [J/mm]	30–60 [7,40]	150–300 [2,21]	200–400 [55]	Proportional to melting enthalpy and heat capacity; Mg lowest due to high thermal conductivity
Required atmosphere	Ar/He [16,39]	He/Ar/vacuum [2,19,45]	He (>99.999%)/vacuum [52,54]	Reactivity progression: Mg oxidation → Ti gas absorption → Zr hydride formation
Required purity [ppm O_2_, N_2_, H_2_]	<100 [39]	<50 [2,19]	<20 (H_2_ < 10) [50,51,52]	Driven by vapor pressure at melting temp (10^5^ → 10^2^ → 10^−1^ Pa) and interstitial reactivity
Main defect mechanism	Evaporation → porosity [7,73]	Gas absorption → brittle martensite [67,184]	Hydride formation → cracking [68,120,200,201]	Vapor pressure hierarchy + slip system limitations
Typical weld hardness [HV]	60–85 [39]	350–450 [67,122]	220–280 [122,123]	Determined by phase transformation kinetics
Cooling rate [K/s]	10^3^–10^4^ [39,80]	10^3^–10^5^ [67,80]	10^2^–10^4^ [121]	Inversely proportional to thermal conductivity (156 → 21.9 → 22.7 W/m·K)
HAZ thickness [mm]	1.5–3.0 [39]	2.0–5.0 [48,114]	1.8–4.5 [55,122]	Proportional to √(κ/ρc_p_); Mg widest due to very high κ
Residual stresses [MPa]	80–150 [180,181]	200–400 [178,179]	150–300 [122,201]	Governed by thermal expansion mismatch (25–27 → 8.6 → 5.7 ×10^−6^/K) and slip system scarcity
Process window width	Moderate [1,7]	Narrow [2,67]	Very narrow [3,50,51,52]	Mg (8–15 combos, ±50%) → Ti (4–6 combos, ±30%) → Zr (2–3 combos, ±10%)
Process window—mechanistic explanation	250 °C evaporation buffer + high thermal conductivity	Low thermal conductivity + phase transformation sensitivity	Highest Tm + contamination limit (<20 ppm) + hydride risk >50 ppm H_2_	-
Linear energy tolerance	±50% (80–400 J/mm) [7,25]	±30% (150–280 J/mm)	±10% (100–200 J/mm) [25,51,52]	Tolerance inversely correlates with process criticality
Relative process cost	1.0× [1]	1.5–2.0× [2,10]	3.0–5.0× [3,50,51,52]	Cost scales with atmosphere purity, monitoring, throughput
Technology readiness level (TRL)	7–8 (production) [1,252]	8–9 (mature) [2,252]	6–7 (pre-commercial) [63,64]	Industrial maturity reflects window width + cost–benefit
Critical property from Table 1	Vapor pressure 10^5^ Pa → evaporation control	Thermal conductivity 21.9 W/m·K → gradients + gas absorption	Melting temperature = 1855 °C + H_2_ sensitivity < 10 ppm + α-Zr expansion 5.7 × 10^−6^/K	-
